# Using Ecological Modeling to Study the Response of Distribution Dynamics of *Paraglenea fortunei* (Coleoptera: Cerambycidae) to Human Activities and Climate Change to in Northeast Asia

**DOI:** 10.1002/ece3.71782

**Published:** 2025-07-08

**Authors:** Ping Wang, Liang Zhang, Jie Li, Chaokun Yang, Guanglin Xie, Wenkai Wang

**Affiliations:** ^1^ Institute of Entomology College of Agriculture, Yangtze University Jingzhou China; ^2^ China National Cotton Research and Development Center, Institute of Industrial Crops Xinjiang Academy of Agricultural Sciences Urumqi China

**Keywords:** climate change, habitat suitability, human activities, *Northeast Asia*, *Paraglenea fortunei*, pest management, species distribution model

## Abstract

Invasive species pose an increasing threat to biodiversity, agriculture, and ecosystem stability, especially under accelerated climate change. *Paraglenea fortunei*, a longhorn beetle native to East Asia, has emerged as a potential pest, warranting urgent attention to its possible range expansion. This study aims to predict the current and future potential distribution of *P. fortunei* using an optimized MaxEnt ecological niche model under various climate change scenarios across Northeast Asia. The results indicate that climatic factors, such as temperature stability, precipitation, and human activities are key drivers influencing its distribution. These findings suggest that *P. fortunei* prefers to live in ecosystems with cooler climates, more consistent changes, and abundant precipitation. Meanwhile, *P. fortunei* may expand to many countries and regions in the future, including central and western China, Sakhalin in Russia, the Hokkaido Islands in Japan, Vietnam, Myanmar, India, Nepal, and Bangladesh. In addition, *P. fortunei* may migrate to higher latitudes as climate conditions change. These findings contribute to a better understanding of climate‐driven distribution dynamics and offer scientific guidance for pest risk management and regional ecological planning.

## Graphical abstract

In recent years, global climate change has become a critical issue affecting various aspects of ecosystems, including the distribution patterns of species. Our study focuses on the distribution dynamics of *Paraglenea fortunei* in Northeast Asia in response to human activities and climate change. The research question we sought to answer was how human activities and climatic factors influence the potential distribution areas and dynamics of *P. fortunei* . This study addresses the gap in the literature regarding the impact of human activities and climate change on insect distribution patterns in the region.

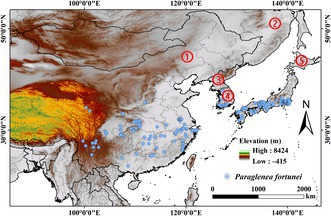

## 1 Introduction

The potential spread of pests can pose a serious threat to forest resources, agricultural productivity, and ecological balance (Wei et al. 2023; Naresh et al. 2024). Understanding how species are distributed under different geographical and environmental conditions, and how these distributions are affected by environmental changes, is crucial for managing pests and maintaining ecosystem stability (Dawson et al. 2011; Pound et al. 2021). As global environmental changes accelerate, pests are likely to expand their ranges, increasing risks to natural ecosystems (Derek and Kyle 2023; Zhang et al. 2024a). Therefore, exploring the relationship between species distribution and environmental variables not only enhances our understanding of pest dynamics, but also provides a scientific basis for implementing targeted control strategies (Lobo et al. 2006; Liu, Liu, et al. 2024; Liu, Peng, et al. 2024; Liu, Zhao, et al. 2024; Zhang et al. 2024b).

Pests are able to damage forest ecosystems not only directly, but also indirectly by destroying plant diversity and altering species composition, thereby affecting the stability of ecosystems and their ability to regulate climate change (Derek and Kyle 2023). Forests are one of the Earth's largest terrestrial ecosystems, which can accumulate organic carbon over time and are an important part of the global carbon cycle, and their ecological security is of great significance for the sustainable development of the natural environment and the socio‐economy (Katri et al. 2024). *Paraglenea fortunei* (Saunders, 1853) (Coleoptera: Cerambycidae) is a phytophagous beetle that is widely distributed in East Asia, including China, North Korea, South Korea, and Japan. As an important stem‐boring pest in Northeast Asia, it not only threatens forest health and the timber industry through its feeding behavior, but also disrupts the ecological balance of the region (Liu et al. 2023). It primarily feeds on *Boehmeria nivea* (L.) Gaudich., 1830, *Hibiscus syriacus* L., 1753, and *Morus alba* L., 1753. Adults feed on the petioles and leaves of host plants, whereas larvae spend their entire developmental life feeding on plant bases and stems, destroying the conductive tissues and leading to stunted growth or even plant death (Togashi 2007). The adults are mostly nocturnal and exhibit a certain degree of phototaxis. Additionally, *P. fortunei* contributes to food web dynamics as prey for predatory insects and birds (Wang et al. 1990). The particular geographical distribution of *P. fortunei* may pose a potential threat to the conservation of forest resources and ecosystem stability in the region, further exacerbating its impact on forest health and agricultural productivity (Kitajima and Makihara 2011). Therefore, understanding the transmission dynamics of *P. fortunei* and its ecological risks is crucial for developing effective pest management strategies.

With increasing global attention on pest management and ecosystem resilience, the compounded effects of human activity and climate change on species distribution are of major concern (Liu, Liu, et al. 2024; Liu, Peng, et al. 2024a; Liu, Zhao, et al. 2024; Zhang et al. 2024c). Forest ecosystems are facing unprecedented challenges, and climate change not only profoundly affects their structural features and functions, but also significantly alters species distribution, abundance, and survival strategies. Therefore, it is important to study the response mechanisms of *P. fortunei* to human activities and climate change and to predict its potential distribution (Kato and Ohbayashi 2010). This study provides an important scientific basis for developing pest management strategies and climate change adaptation measures in Northeast Asia, and reveals potential transmission trends and management strategies for *P. fortunei* .

Species distribution models (SDMs) are modeling and analytical tools based on the theory of actual ecological niches. They are widely used in the fields of species ecology, conservation biology, and environmental health management, including biodiversity assessment, protected area planning, invasive species management, disease transmission prediction, and ecological restoration and rehabilitation (Jung et al. 2024; Zhang et al. 2024d). Predicting the potential distribution of species in unobserved areas by analyzing the relationship between known species distribution points and environmental variables can provide a basis for ecological risk assessment and the development of management strategies (Sacha et al. 2019). Similar studies have used SDMs to predict the potential distribution of alien species globally and have provided important references for invasive species management. For example, Zhou et al. (2022) applied the CLIMEX and MaxEnt models to predict the potential distribution of *Anoplophora chinensis* and analyzed the impact of climate change on its distribution. Zhao et al. (2024) used the maximum entropy model to predict the potential distribution of 35 invasive alien mealybugs aiming to help develop invasive species prevention and control measures in China.

In this study, we applied an optimized MaxEnt ecological niche model (ENM) to analyze the current distribution patterns of *P. fortunei* in Northeast Asia and to project its potential future distribution under changing environmental conditions. Our objectives are: (1) to identify key climatic factors affecting the distribution of *P. fortunei* ; (2) to compare the current habitat distribution of *P. fortunei* with and without anthropogenic disturbance; and (3) to predict the potential future distribution of *P. fortunei* under different climate change scenarios. This study aims to reveal the interrelationships between the distribution pattern of *P. fortunei* and environmental factors, to provide a theoretical basis for regional quarantine management, early warning, and sustainable control strategies in regions where the species has not yet established. Furthermore, the study explores the impact of climate change on its dispersal mechanisms, offering valuable insights for forest pest management, ecosystem protection, and forest resource management in Northeast Asia.

## 2 Methods

### 2.1 Data Source

#### 2.1.1 Research Area

This study aimed to assess the potential habitat distribution of *P. fortunei* , a species currently confined to East Asia. Specifically, *P. fortunei* is mainly distributed in China, North Korea, South Korea, and Japan. All the species distribution data extracted and environmental variables analyzed in the study were restricted to these four countries and their surrounding regions. By constructing a climate suitability model, this study predicts the potential distribution pattern and ecological adaptive capacity of *P. fortunei* in East Asia at a regional scale, thereby providing a scientific basis for the management and early warning of this species in the context of climate change.

#### 2.1.2 Species Distribution Data

**FIGURE 1:**
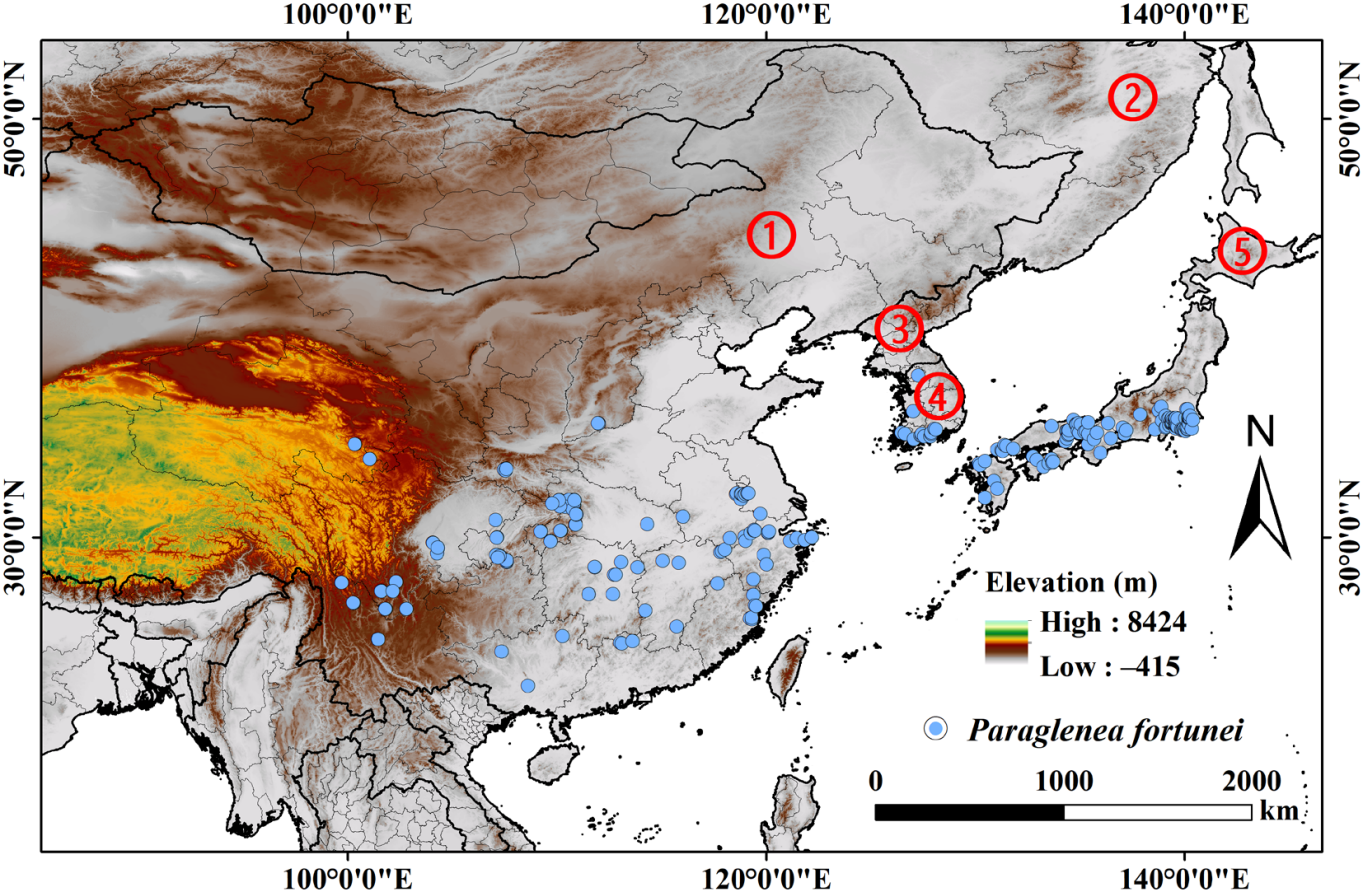
Occurrence records of *P. fortunei* within Northeast Asia. ①: China; ②: Russia; ③: North Korea; ④: South Korea; ⑤: Japan.

The sources of data on the distribution of *P. fortunei* in this study were diverse, including: (1) book materials and online references (CNKI, https://www.cnki.net/, accessed on June 22, 2024; WOS, https://www.webofscience.com/wos, accessed on June 23, 2024; NACRC, http://museum.ioz.ac.cn/, accessed on June 24, 2024); (2) two online public databases, Global Biodiversity Information Facility (https://doi.org/10.15468/dl.g3ryqj, accessed on June 23, 2024) and iNaturalist (https://www.inaturalist.org/, accessed on June 24, 2024); and (3) Laboratory personnel obtained records of *P. fortunei* occurrences during field surveys conducted from 2013 to 2024. For distribution points lacking specific latitude and longitude coordinates in the original records, online Google Earth tools (http://ditu.google.cn/, accessed on June 27, 2024) were used to obtain this critical geographic information (Zhang et al. 2021; Zhang et al. 2024b). We collected occurrence records for over 1800 species from multiple sources. We excluded data points with inaccurate geographic coordinates or incomplete information, and also removed duplicate records. After this initial screening, 502 species occurrence records were retained (Figure 1).

To avoid overfitting of the model due to spatial autocorrelation, we sparsified the data using the “ENMTools” package (version 1.1.3) of R software (version 4.3.1) platform (Warren and Seifert 2011; Zhang et al. 2024c). The sparse distance was set to 2.5 arc‐minutes (~4.6 km), which corresponds to the size of the bioclimatic data grid cells to eliminate redundant data recorded multiple times in the same grid (Warren et al. 2021). The filtered final data ensured the quality of the dataset and reduced potential overfitting bias due to spatial aggregation, thereby improving the predictive accuracy and robustness of the model. Through screening, 240 distribution records were eventually retained for modeling.

#### 2.1.3 Environmental Data

Climate data were downloaded from WorldClim version 2.1 (https://www.worldclim.org/, were accessed on January 24, 2024). We obtained climate data for the current period (1970–2000) and for two future periods (2050s and 2070s). Future climate data were selected from the Beijing Climate Centre Climate System Model 2 Moderate Resolution (BCC‐CSM2‐MR) climate system model from the Sixth International Coupled Model Intercomparison Programme Phase 6 (Wu et al. 2019). These variables include key climate factors such as temperature, precipitation, and humidity, and provide a comprehensive picture of species' responses to environmental change. These datasets include 19 bioclimatic variables with a resolution of 2.5 arc‐minutes (Wu et al. 2019). For future climate scenarios, we used the BCC‐CSM2‐MR global circulation model, which is able to simulate the interactions of the atmosphere, oceans, land, and sea ice to project future climate change. We selected future climate data for 2041–2060 (2050s) and 2061–2080 (2070s) and combined them with four shared socioeconomic pathway (SSP) scenarios: Low forcing scenario (SSP1‐2.6), medium forcing scenario (SSP2‐4.5 and SSP3‐7.0), and a high forcing scenario (SSP5‐8.5). These scenarios are based on different socioeconomic development pathways, modeling potential future global impacts in terms of greenhouse gas emissions, socioeconomic development, and climate change, respectively, and providing a variety of possibilities for predicting the future suitable distribution of *P. fortunei* (Fick and Hijmans 2017). We downloaded human activity data, including the global human impact index (GHII) and the global human footprint (GHF), from the Socioeconomic Data and Applications Center (https://sedac.ciesin.columbia.edu, accessed on 15 January 2024). In addition, we downloaded population density (PD) data from the worldpop (https://hub.worldpop.org, accessed on 15 August 2024) database. Finally, we used the “Resample” and “Extract” tools in the ArcGIS Map (version 10.8.1) software to standardize all 22 environmental variables (both bioclimatic and anthropogenic) to have the same spatial extent and resolution (2.5 arc‐minutes) for subsequent analysis and modeling.

Multicollinearity among environmental variables can affect model prediction accuracy, making it essential to analyze the correlations between variables before model prediction (Hadi Ahmad et al. 2023). Initially, the contribution of 22 environmental variables in the MaxEnt model (version 3.4.1) was assessed through the Jackknife method as a way of determining their importance in the model predictions. Subsequently, Pearson correlation analysis was conducted on these 22 environmental variables using the “ENMTools” package in R (Figure S1). When the correlation coefficient |*r*| between any two climate factor variables exceeds 0.9, it indicates a very strong linear association. To avoid the problem of multicollinearity, the variable that contributes more to the explanatory power of the model should be selected for inclusion in the analysis. After screening, the mean diurnal range (Bio2), isothermality (Bio3), temperature seasonality (Bio4), mean temperature of warmest quarter (Bio10), mean temperature of coldest quarter (Bio11), precipitation of driest month (Bio14), precipitation seasonality (Bio15), precipitation of warmest quarter (Bio18), GHII, GHF, and PD were finally retained for model construction (Table S1).

### 2.2 MaxEnt Model Evaluation and Validation

**FIGURE 2:**
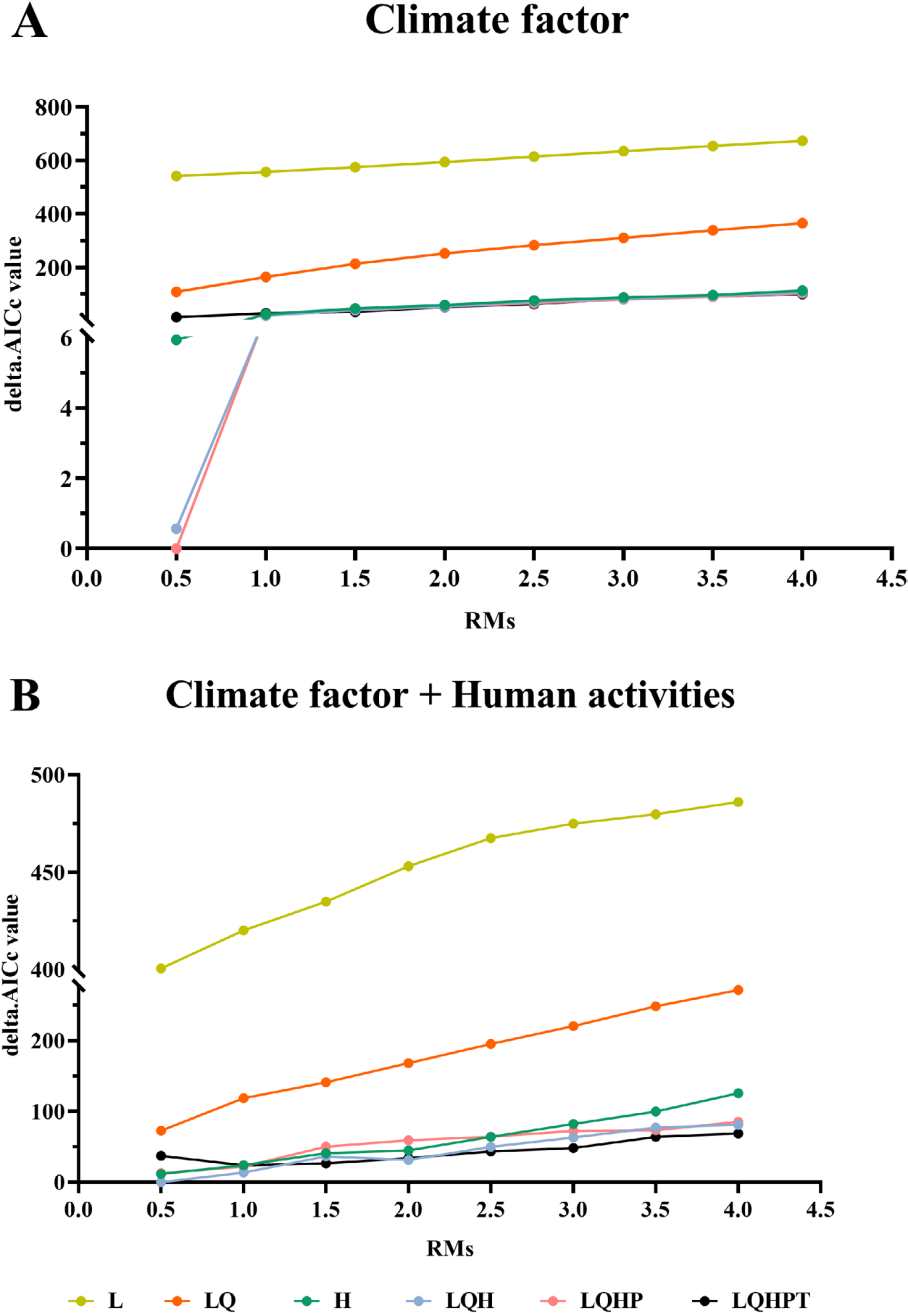
Calculated delta.AICc values for different FCs combinations based on the ENMeval package. (A) Climate factor; (B) Climate factor + human activities.

The MaxEnt model is one of the main methods used in ENMs to construct models for prediction by maximizing entropy, that is, selecting the most homogeneous and unbiased data to simulate the potential distribution of a species in the presence of incomplete data (Wang et al. 2019). In the MaxEnt model, the regularization multiplier (RM) and feature combinations (FCs) are crucial parameters, significantly impacting the predictive accuracy of the model (Li et al. 2022). Using the “ENMeval” (version 2.0.4) package in R, these parameters were meticulously optimized (Warren et al. 2021). The FCs encompass five types: L (linear), Q (quadratic), P (product), T (threshold), and H (hinge). The RM was set from 0 to 4 with an interval of 0.5, based on retained distribution data points and climatic selected environmental variables. Six FCs were specified to explore the optimal parameter combination: L, LQ, H, LQH, LQHP, and LQHPT. Consequently, 48 different RM and FC combinations were constructed. The AICc value was used as the criterion to determine the optimal model, selecting the model with the smallest deltaAICc value. Results indicated that setting RM to 0.5 and FCs to “LQHP” yielded a delta.AICc value of 0 (Figure 2). Other parameter settings for the optimal model include using 25% of distribution points for testing and 75% for training, a maximum iteration number of 5000, a limit of 10,000 background points, and 10 replications. The proximity of the test omission rate to the theoretical omission rate was considered a critical indicator of model accuracy (Phillips et al. 2006). This series of refined parameter optimizations and model construction aimed to ensure the MaxEnt model's highest accuracy and reliability in predicting the potential distribution of *P. fortunei* , thereby providing a scientific basis for ecological conservation and natural resource management.

The receiver operating characteristic curve (ROC) and its area under the curve (AUC) are key indicators for assessing model accuracy and predictive performance (Kaky et al. 2020). The AUC value ranges from 0 to 1, directly reflecting the model's predictive precision. Specifically, a higher AUC value indicates higher predictive accuracy. Although AUC value is widely used in SDMs, it is not the only criterion for assessing model accuracy. Previous studies have criticized the shortcomings of AUC value due to the fact that it assigns equal weight to omission errors and commission errors (Liu et al. 2022). With this in mind, we also chose the true skill statistic (TSS), defined as “sensitivity + specificity −1”, as the relevant metric for assessing the accuracy of the model. Performance is not better than random. Based on the predictions of the maxent model and the actual distribution points, we used the “PresenceAbsence” package (version 1.1.11) to calculate the TSS values (Freeman and Moisen 2008). With these key evaluation criteria, we are able to fully understand the performance of the model and make the necessary adjustments based on the evaluation results to improve the overall performance of the model and the accuracy of the predictions.

### 2.3 Changes in the Potential Distribution Areas of *P. fortunei*

The effects of bioclimatic variables and human activities on the geospatial distribution pattern of *P. fortunei* were investigated by modeling three different scenarios: (1) prediction based on current bioclimatic variables (eight in total); (2) prediction based on current bioclimatic variables and human activities (including GHII, GHF, and PD); (3) prediction based on future bioclimatic variables. Models (1) and (2) are predictions made based on current climate models, and model (3) is a prediction based on future climate models. Models (1) and (2) were used to assess anthropogenic impacts on habitat suitability of *P. fortunei* , whereas models (1) and (3) were used to explore potential impacts of climate change on habitat suitability of *P. fortunei* .

To enhance the accuracy and reliability of the MaxEnt model, a repeated run strategy was employed (Gao et al. 2023). Specifically, the optimal MaxEnt model was run 10 times using the logistic output for species presence probability (*p*), and the average of these runs was used as the final prediction. This approach helps reduce the random variability in model outputs, thereby increasing the stability and credibility of the predictions. The results were then converted into raster format and visualized using ArcGIS Map to more intuitively display the potential distribution areas of *P. fortunei* .

According to the Intergovernmental Panel on Climate Change (IPCC) report, the suitability of habitats for *P. fortunei* was classified into four categories (Gao et al. 2023): Unsuitable habitat (*p* < 0.05), Low suitability habitat (0.05 ≤ *p* < 0.33), Moderately suitable habitat (0.33 ≤ *p* < 0.66), and Highly suitable habitat (0.66 ≤ *p* ≤ 1). The area proportions of each suitability category were calculated by counting the number of raster cells in each category, and the data were clearly presented using Graphpad Prism (version 9.0) software.

In our study, we utilized the “Distribution Changes between Species Distribution Models (SDMs)” feature in SDMToolbox (version 2.6), we were able to thoroughly assess the relative dynamics of *P. fortunei* 's distribution under various future climate change scenarios (Aidoo et al. 2022; Yang et al. 2022). In this process, a threshold of 0.05 was set to generate binary maps, which were then compared with binary maps under current climate scenarios to reveal potential changes in species distribution. The study results can be categorized into four types of changes: (1) Expansion: The species' distribution range significantly expands under future climate conditions. (2) No Occupancy: The species does not occupy any new distribution areas under future scenarios. (3) Unchanged: The species' range remains stable under different future climate scenarios. (4) Contraction: The species' distribution range significantly shrinks. These results not only accurately reflect the potential distribution changes of *P. fortunei* under different climate change scenarios but also provide robust support for a deeper analysis of the species' response mechanisms to climate change.

### 2.4 Change of Potential Distribution Center Shift Under Future Climate Scenarios

Using the “Centroid Changes (Lines)” function in “SDMToolbox” (version 2.6), the potential geographic distribution center of *P. fortunei* under various future carbon emission scenarios was analyzed (Zhang, Dang, et al. 2022; Zhang, Wang, et al. 2022; Yoon and Lee 2023). This tool quantifies the temporal shifts in the species' distribution center and visually links the potential distribution centers across different periods. This method enables an intuitive observation of how the distribution center of *P. fortunei* changes over time, allowing for the identification of the spatial trajectory of its primary suitable growth areas under different future climate scenarios.

## 3 Results

### 3.1 Model Accuracy and Evaluation

This study evaluates the accuracy of the optimized MaxEnt model in predicting the distribution of *P. fortunei* using test AUC and test TSS values. Specifically, when considering only climatic factors, the optimized model achieves an average AUC value of 0.981 and a TSS of 0.813. The average AUC value was 0.976 and the TSS value was 0.817 after the inclusion of anthropogenic activity‐related variables (including global human influence index, GHF, PD). Meanwhile, the sensitivity was higher than 0.79 and the specificity was 0.87. This indicates that the optimized MaxEnt model is able to distinguish well between the actual distribution and nondistribution areas of *P. fortunei* in the current period. Furthermore, under various future climate change scenarios, the model consistently achieves an average AUC value above 0.96 and TSS value above 0.71, further affirming its robustness and generalization capacity (Table 1). The actual omission rate curves were highly consistent with the expected omission rate curves, especially in the low threshold range, where the two almost overlapped, indicating that the model has strong predictive ability and low risk of overfitting (Figure S2). In addition, the deviation between the two curves was small at most thresholds, indicating that the model has good stability and reliability in predicting the suitability areas of *P. fortunei* . These findings suggest that the model not only distinguishes well between current distributions, but also maintains a high level of discriminatory accuracy under future climate change scenarios.

**TABLE 1:** Model accuracy evaluation.

Parameters	Shared socioeconomic pathways	Training AUC (average value)	Test AUC (average value)
Default	Current‐climate factor	0.9891	0.9884
	Curren‐climate factor + human activities	0.9826	0.9755
Optimize	Current‐climate factor	0.9898	0.9807
	Curren‐climate factor + human activities	0.9898	0.9885
	Future‐SSP1‐2.62041–2060	0.9711	0.9706
	Future‐SSP1‐2.62061–2080	0.9606	0.9604
	Future‐SSP2‐4.52041–2060	0.9710	0.9705
	Future‐SSP2‐4.52061–2080	0.9813	0.9809
	Future‐SSP3‐7.02041–2060	0.9609	0.9606
	Future‐SSP3‐7.02061–2080	0.9912	0.9907
	Future‐SSP5‐8.52041–2060	0.9710	0.9708
	Future‐SSP5‐8.52061–2080	0.9710	0.9704

### 3.2 Environmental Variable Analysis

**FIGURE 3:**
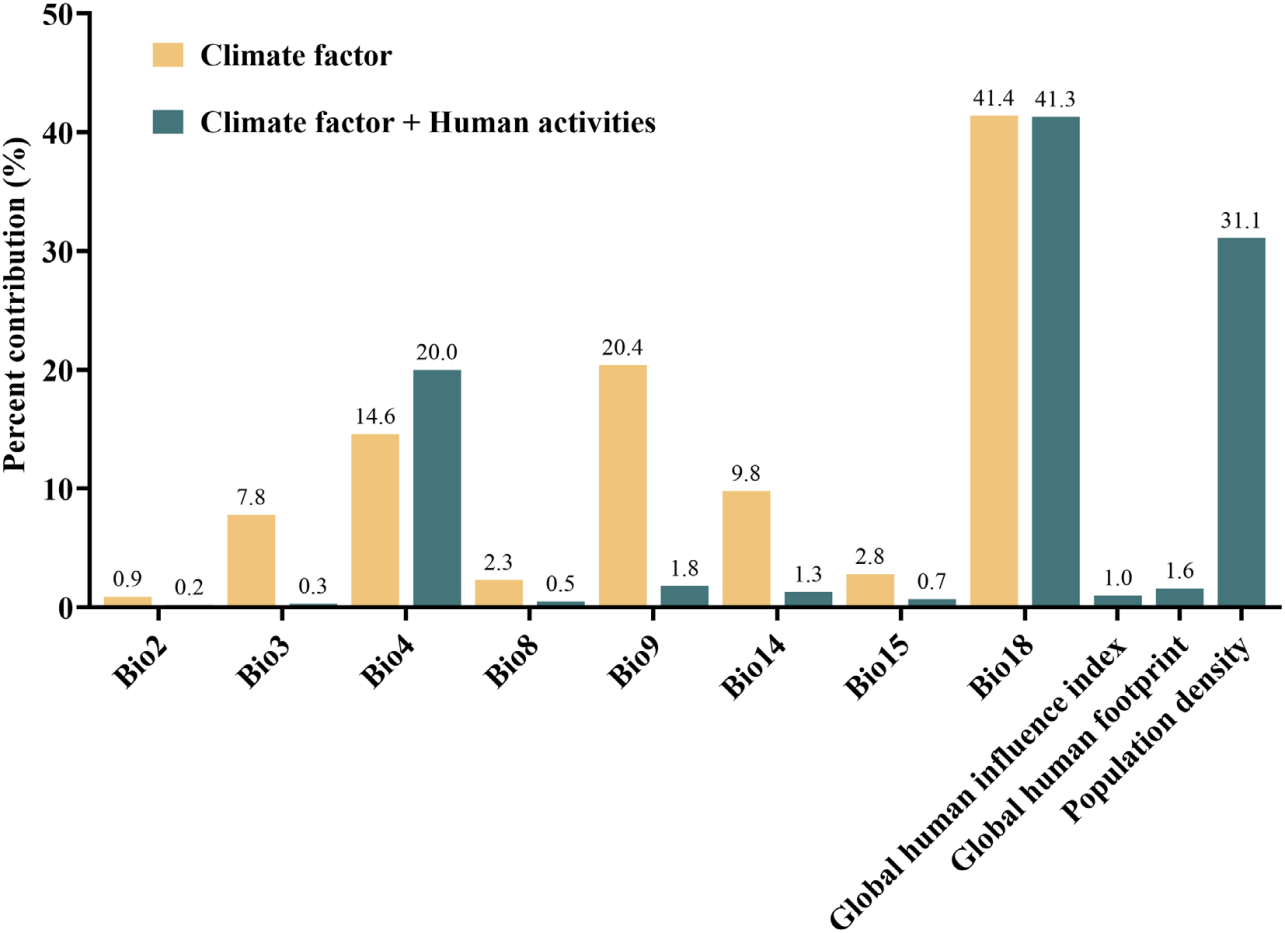
The contribution rates of the environmental variables in MaxEnt model.

The results indicate that precipitation of the warmest quarter (Bio18, 41.4% ± 4.68%) has the greatest contribution to the distribution of *P. fortunei* , followed by mean temperature of the driest quarter (Bio9, 20.4%), temperature seasonality (Bio4, 14.6%), and precipitation of the driest month (Bio14, 9.8%), with isothermality (Bio3, 7.8%) having the least contribution. The cumulative contribution rate of these variables is 94% (Figure 3). This suggests that the distribution of *P. fortunei* is influenced by multiple factors, including temperature, precipitation, and seasonality, highlighting the significant role of these climatic variables in explaining the distribution patterns of the species. When considering anthropogenic factors, the main factors affecting the geographical distribution of *P. fortunei* are temperature seasonality (Bio4, 20%), precipitation of the warmest quarter (Bio18, 41.3%), and population density (PD, 31.1%). This indicates that in addition to bioclimatic factors, human activities also play a significant role in the distribution of *P. fortunei* .

**FIGURE 4:**
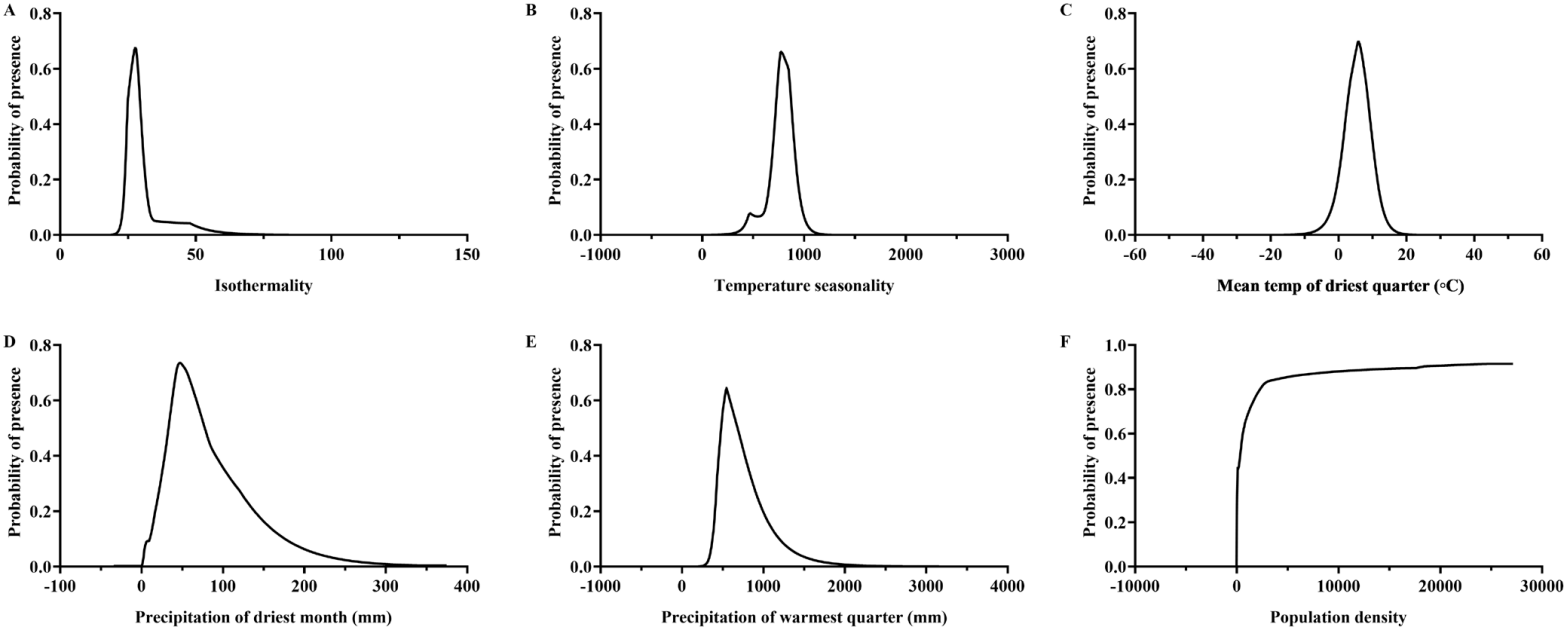
Response of environmental factors. (A) Bio3; (B) Bio4; (C) Bio9; (D) Bio14; (E) Bio18; (F) Bio22.

Furthermore, Figure 4 illustrates the response curves between the probability of presence of *P. fortunei* and key environmental variables related to its distribution. Following the methodology of Xian et al. (2023), the evaluation was conducted within a probability range > 0.4. The results indicate that the potential distribution probability of *P. fortunei* peaks at 67.44% when isothermality (Bio3) is 27.81. Isothermality refers to the ratio of day‐to‐night temperature oscillation relative to the annual temperature range. A value of 27.81 suggests that *P. fortunei* is most likely to thrive in areas with moderate diurnal temperature variation and relatively stable temperature regimes. Such thermal stability may support physiological processes such as development and reproduction, which are crucial for the establishment of populations. Similarly, the potential distribution probability peaks at 66.07% when temperature seasonality (Bio4) reaches 773.30, with relatively high probabilities maintained between 713.74 and 886.74. This suggests that *P. fortunei* can tolerate a certain degree of temperature variability across seasons, which may facilitate its expansion into diverse climatic zones (Figure 4). Additionally, the potential distribution probability is highest at 69.71% when the mean temperature of the driest quarter (Bio9) is 5.73°C, with a relatively high probability also observed between 1.88°C and 9.40°C. This indicates a preference for relatively cool but not freezing dry‐season conditions. Notably, the potential distribution probability of *P. fortunei* shows an initial increase followed by a decrease with increasing precipitation of the driest month (Bio14) and precipitation of the warmest quarter (Bio18) values. Specifically, the probability peaks at the precipitation of the driest month (Bio14) of 46.92 mm, remaining high between 28.51 and 91.12 mm. Likewise, the probability peaks at the precipitation of the warmest quarter (Bio18) of 546.45 mm, remaining high between 453.46 and 770.31 mm (Figure 4). These results suggest that *P. fortunei* favors moderately moist conditions but may be limited in very arid or extremely wet environments. Notably, the response to anthropogenic factors, especially population density (PD), shows a clear positive trend. The potential distribution probability of *P. fortunei* increases with rising human population density. This implies that human activities such as urban development, transportation, and trade may inadvertently facilitate the spread of *P. fortunei* , either by creating microhabitats or through passive dispersal mechanisms. This finding aligns with other studies highlighting the role of human disturbance in the success of invasive species.

### 3.3 Potential Geographical Distribution Area of *P. fortunei* Under Current Climate Conditions

**FIGURE 5:**
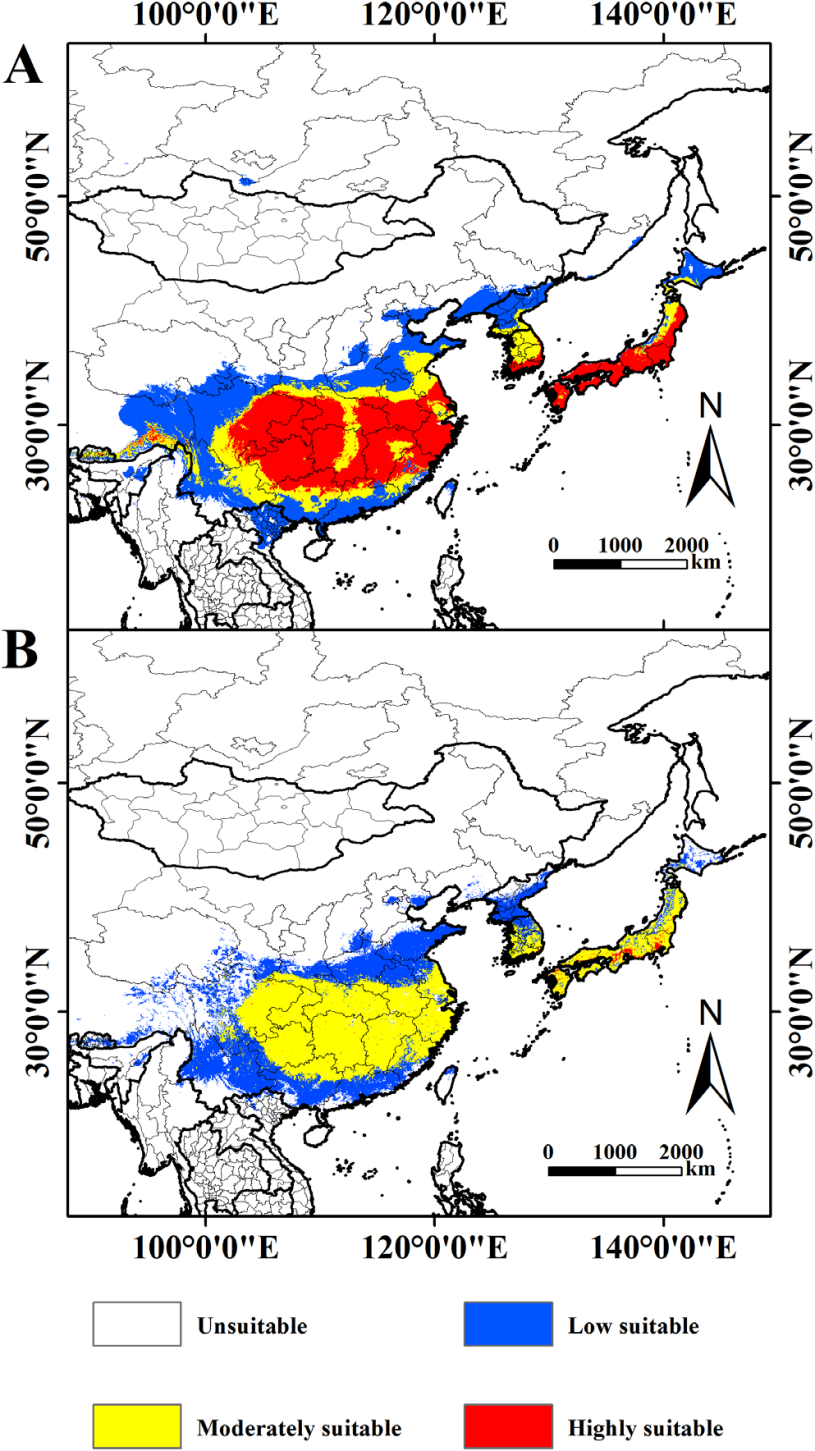
Distribution of suitable habitat for *P. fortunei* in Northeast Asia under current climate models.

The ENM (MaxEnt) was used to predict the potential geographic distribution of *P. fortunei* , revealing that the species is primarily distributed across East Asia, which aligns closely with its actual occurrence (Figure 5). This confirms the high accuracy of the optimized MaxEnt model in predicting the potential distribution areas of *P. fortunei* . Under current climatic conditions, when only climatic factors are considered, the potential distribution area of *P. fortunei* mainly covers central and southern China, central and southern Japan, southern South Korea, southern North Korea, northeastern Vietnam, and central Bhutan and Nepal. The total suitable habitat area is approximately 413.46 × 10^4^ km^2^, representing 0.28% of the world's land area. Among this, the high suitability habitat covers 147.16 × 10^4^ km^2^, accounting for 35.59% of the total suitable habitat. The medium suitability habitat covers 89.35 × 10^4^ km^2^, representing 21.61% of the total suitable habitat, whereas the low suitability habitat totals 176.95 × 10^4^ km^2^, making up 42.80% of the total suitable habitat (Table 2). Considering the anthropogenic factor, the total area of suitable habitat for *P. fortunei* in Northeast Asia was 314.88 × 10^4^ km^2^. Among them, the area of highly suitable habitat was 2.73 × 10^4^ km^2^, accounting for 0.87% of the total area of suitable habitat. The area of medium suitable habitat was 161.18 × 10^4^ km^2^, accounting for 51.19% of the total suitable habitat area, and the area of low suitable habitat was 150.97 × 10^4^ km^2^, accounting for 47.95% of the total suitable habitat area. Compared with the area affected only by natural environmental factors, the total area of suitable habitat for *P. fortunei* decreased by 98.58 × 10^4^ km^2^, indicating that human activities have reduced the area of suitable habitat for *P. fortunei* .

**TABLE 2:** Predicted suitable areas for *Paraglenea fortunei* under current and future climatic conditions.

Shared socioeconomic pathways	Predicted area (10^4^ km^2^)			Comparison with current distribution (%)		
	Low suitable	Medium suitable	High suitable	Low suitable	Medium suitable	High suitable
Current‐climate factor	176.95	89.35	147.16	—	—	—
Current‐climate factor + human activities	150.97	161.18	2.73	—	—	—
Future‐SSP1‐2.62041–2060	203.17	101.19	138.89	14.82	13.26	−5.62
Future‐SSP1‐2.62061–2080	166.01	95.35	149.52	−6.18	6.72	1.60
Future‐SSP2‐4.52041–2060	192.03	109.64	155.09	8.53	22.72	5.39
Future‐SSP2‐4.52061–2080	174.73	99.33	141.68	−1.25	11.17	−3.73
Future‐SSP3‐7.02041–2060	199.40	88.63	144.45	12.69	−0.80	−1.85
Future‐SSP3‐7.02061–2080	182.78	83.91	149.96	3.29	−6.08	1.90
Future‐SSP5‐8.52041–2060	175.24	96.72	146.96	−0.97	8.25	−0.14
Future‐SSP5‐8.52061–2080	207.08	70.11	161.20	17.03	−21.53	9.54

### 3.4 Potential Geographical Distribution Area of *P. fortunei* Under Different Climate Scenarios in the Future

**FIGURE 6:**
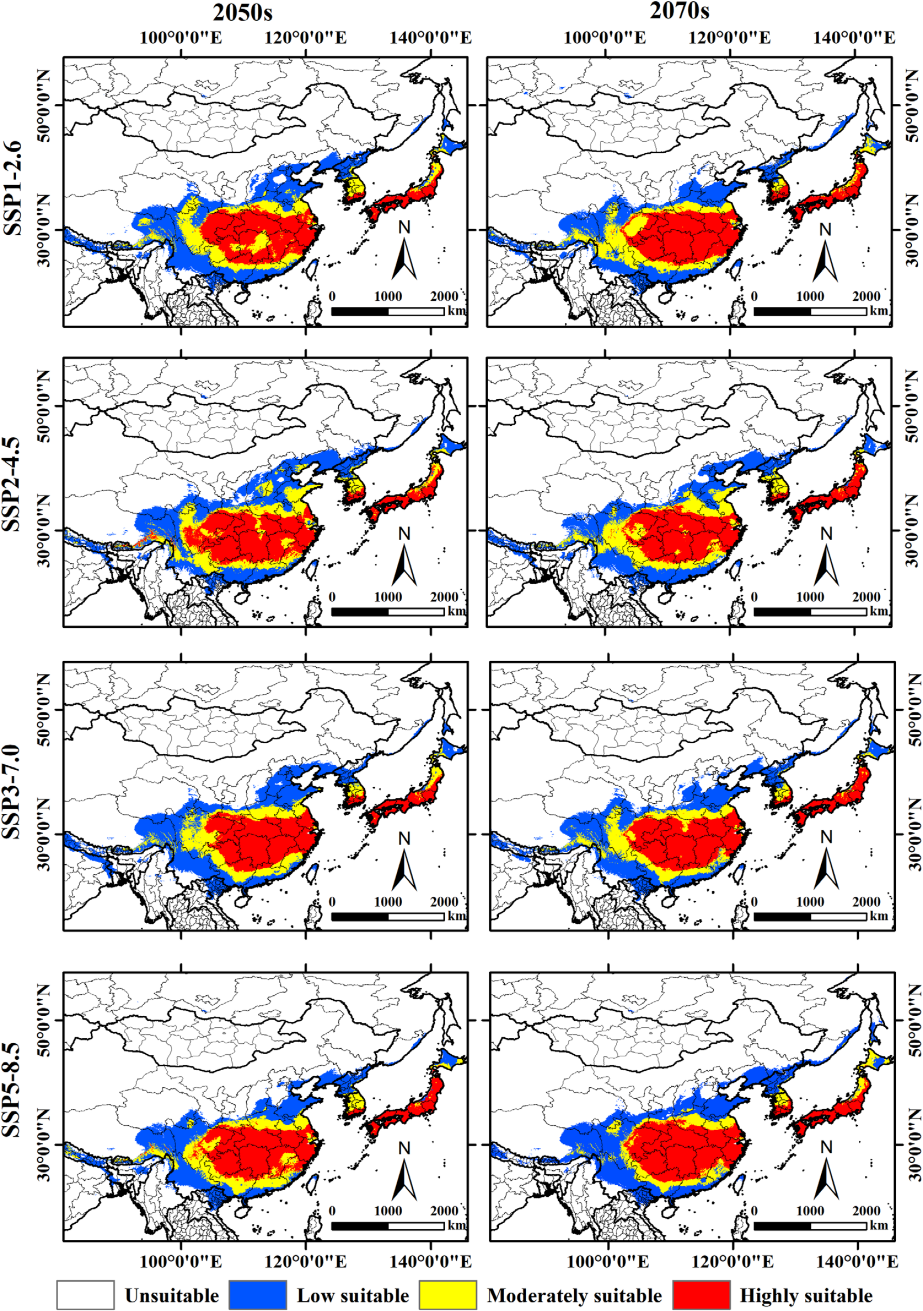
Suitable habitat distribution of *P. fortunei* in Northeast Asia under different climate models in the future.

Under different future climate scenarios, the projected suitable habitat range for *P. fortunei* remains largely consistent with the current distribution, primarily encompassing China, North Korea, South Korea, Japan, Vietnam, Bhutan, India, and Nepal (Figure 6). The results indicate that, despite potential future climatic changes, the ecological requirements and adaptability of *P. fortunei* may result in a relatively stable range of suitable habitats. In future climate scenarios, the total area of suitable habitat for *P. fortunei* is projected to range from 410.88 × 10^4^ km^2^ to 456.76 × 10^4^ km^2^, which represents 0.28%–0.31% of the world's land area (Table 2). The area of suitable habitat for *P. fortunei* varies between scenarios, with SSP2‐4.5‐2050s showing the largest suitable habitat area, followed by SSP1‐2.6‐2050s, and SSP1‐2.6‐2070s predicting the smallest suitable habitat area. Except for the SSP1‐2.6‐2070s scenario, where the suitable habitat area is smaller than at present, the suitable habitat areas in all other future scenarios are larger than the current area, suggesting that *P. fortunei* may find more suitable habitats under future climate conditions. Over time, under low greenhouse gas emission scenarios (SSP1‐2.6, SSP2‐4.5) and moderate greenhouse gas emission scenarios (SSP3‐7.0), the suitable habitat area shows an initial increase followed by a decrease, with an increase in the 2050s and a decrease in the 2070s.

### 3.5 Relative Changes of Potential Distribution Area of *P. fortunei* Under Different Climate Scenarios in the Future

**FIGURE 7:**
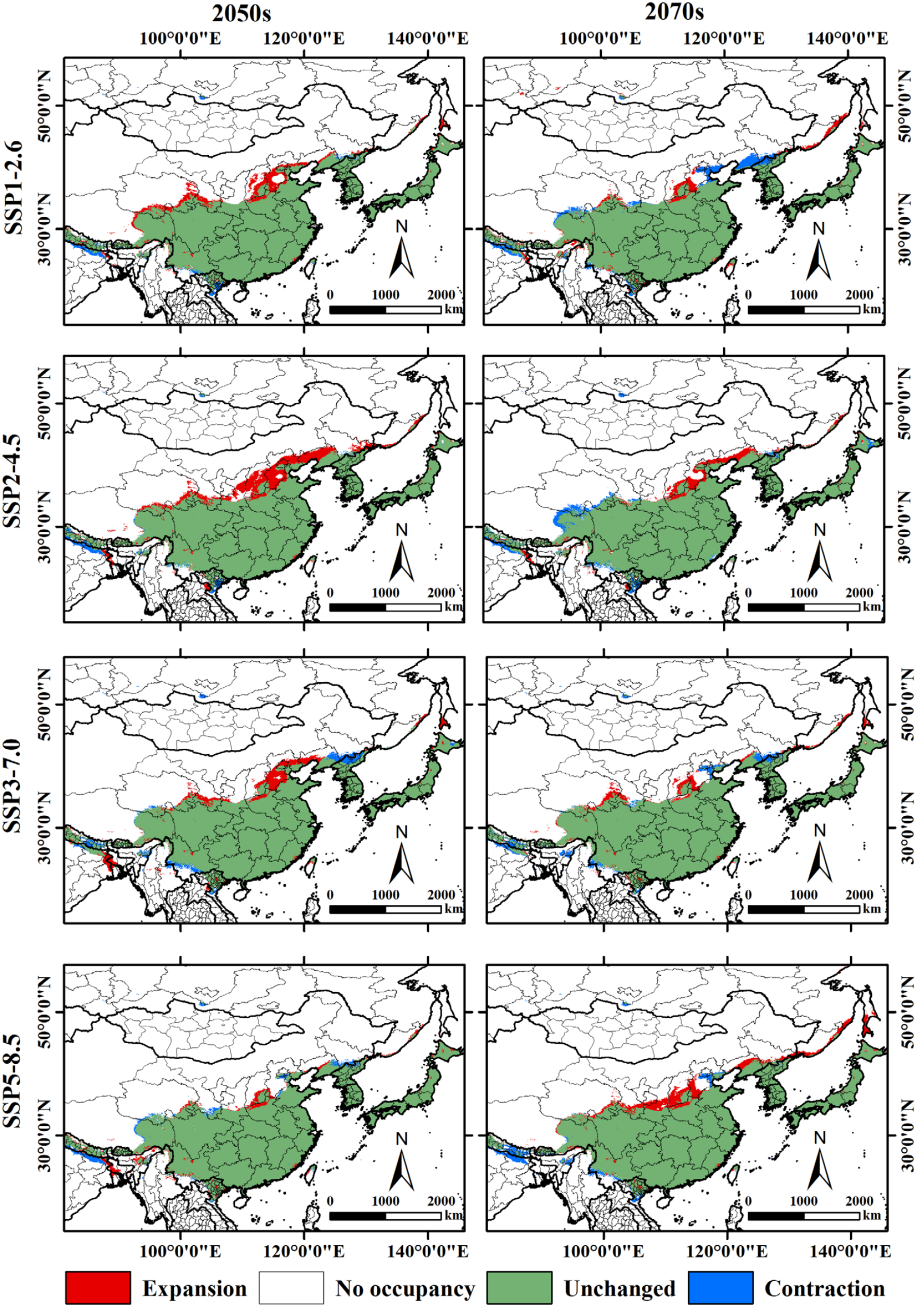
Changes of potential suitable habitat of *P. fortunei* in Northeast Asia under different climate models in the future.

By comparing the current and future distribution patterns, we assessed the relative changes in the suitable habitat range of *P. fortunei* under different future climate scenarios. The results indicate that *P. fortunei* may undergo both expansion and contraction of suitable habitat (Figure 7). The expansion areas range from 18.16 to 29.82 × 10^4^ km^2^, whereas contraction areas range from 6.71 to 14.39 × 10^4^ km^2^ (Table 3). Habitat expansion is projected mainly in central and western China, Russia, Japan, Vietnam, Myanmar, India, Nepal, and Bangladesh. In contrast, habitat contraction is mainly observed in eastern and southwestern China, Russia, North Korea, Japan, Vietnam, Myanmar, Bangladesh, India, and Nepal. The extent of expansion varies across future climate scenarios, with the largest expansion predicted under the high‐emission scenario SSP5‐8.5 in the 2070s, followed by SSP2‐4.5 in the 2050s, and the smallest under SSP2‐4.5 in the 2070s. The greatest contraction is projected under SSP2‐4.5 in the 2070s, whereas the smallest contraction occurs under SSP1‐2.6 in the 2070s (Table 3). These findings underscore the significant influence of climate change and socioeconomic development pathways on the future habitat suitability of *P. fortunei* .

**TABLE 3:** Predicted suitable areas for *P. fortunei* under current and future climatic conditions.

Shared socioeconomic pathways	Predicted area (10^4^ km^2^)			
	Expansion	No occupancy	Unchanged	Contraction
Future‐SSP1‐2.6‐2041–2060	75.16	64157.53	519.54	47.77
Future‐SSP1‐2.6‐2061–2080	95.16	64137.53	521.89	45.42
Future‐SSP2‐4.5‐2041–2060	97.90	64134.79	505.80	61.51
Future‐SSP2‐4.5‐2061–2080	46.14	64186.55	477.23	90.07
Future‐SSP3‐7.0‐2041–2060	78.38	64154.31	509.80	57.51
Future‐SSP3‐7.0‐2061–2080	67.00	64165.69	488.69	78.62
Future‐SSP5‐8.5‐2041–2060	49.58	64183.11	491.53	75.78
Future‐SSP5‐8.5‐2061–2080	119.49	64113.20	508.39	58.92

### 3.6 Spatial Change Route of *P. fortunei* Under Different Climate Scenarios in the Future

**FIGURE 8:**
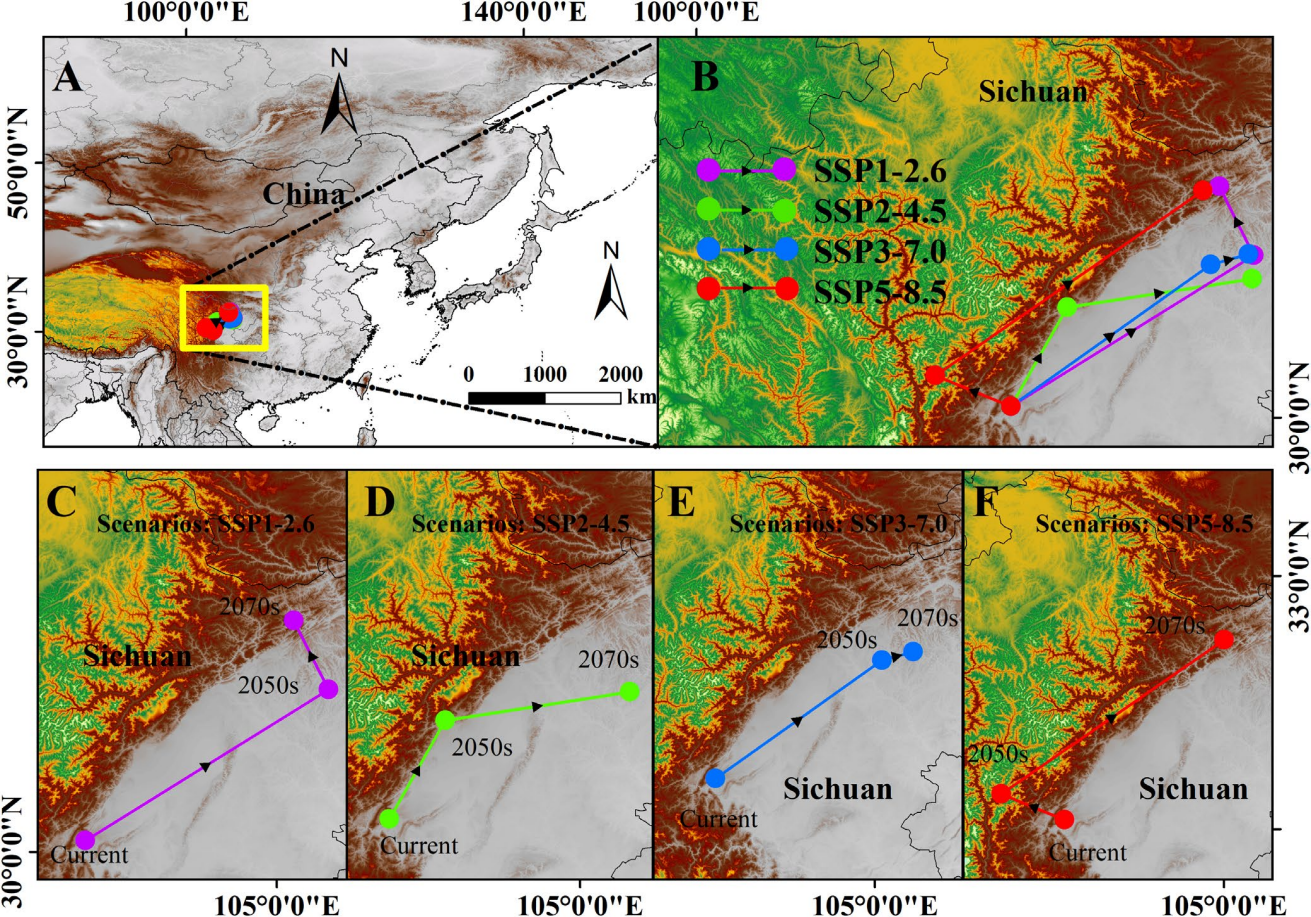
(A) Potential regional spatial change trajectory of *P. fortunei* in Northeast Asia. (B) Spatial change path of *P. fortunei* under four different types of future climate scenarios; (C) SSP1‐2.6; (D) SSP2‐4.5; (E) SSP3‐7.0; (F) SSP5‐8.5.

The projected migration of the potential geographic distribution center of *P. fortunei* reveals a trend of shifting toward higher latitudes under different future climate scenarios (Figure 8). In the current period, the center of distribution of *P. fortunei* is located in Sichuan Province, China (31.60° N, 113.18° E). Under future climate scenarios, the potential distribution center of *P. fortunei* remains in Sichuan Province, China (Figure 8). Specifically, under the SSP1‐2.6 scenario, the distribution center in the 2050s is at 31.60° N, 105.51° E, and in the 2070s at 32.28° N, 105.16° E. For the SSP2‐4.5 scenario, the distribution center in the 2050s is at 31.09° N, 103.66° E, and in the 2070s at 31.37° N, 105.49° E. In the SSP3‐7.0 scenario, the 2050s distribution center is at 31.51° N, 105.08° E, and the 2070s center is at 31.61° N, 105.45° E. Under the SSP5‐8.5 scenario, the 2050s distribution center is at 30.41° N, 102.36° E, and the 2070s center is at 32.24° N, 105.00° E (Table 4). These results suggest that under different future greenhouse gas emission scenarios, *P. fortunei* may migrate to regions with higher latitudes. This reflects the potential for *P. fortunei* to further adapt to climate change and seek new suitable habitats under future carbon reduction scenarios.

**TABLE 4:** Longitude, latitude, and migration distance of *P. fortunei* in different periods.

Shared socioeconomic pathways	Longitude (°E)	Latitude (°N)	Center migration distance (km)
Current	103.11	30.11	—
Future‐SSP1‐2.6‐2041–2060	105.51	31.60	282.91
Future‐SSP1‐2.6‐2061–2080	105.16	32.28	310.70
Future‐SSP2‐4.5‐2041–2060	103.66	31.09	121.02
Future‐SSP2‐4.5‐2061–2080	105.49	31.37	267.71
Future‐SSP3‐7.0‐2041–2060	105.08	31.51	244.49
Future‐SSP3‐7.0‐2061–2080	105.45	31.61	279.39
Future‐SSP5‐8.5‐2041–2060	102.36	30.41	79.61
Future‐SSP5‐8.5‐2061–2080	105.00	32.24	297.46

## 4 Discussion

The *P. fortunei* , as a typical wood‐boring pest widely distributed in East Asia, is likely to further expand its distribution pattern and range of activities with the intensification of climate change, which will significantly increase its invasion risk and colonization pressure, posing a serious threat to forestry and agroecosystems (Wang et al. 1990; Togashi 2007). However, with future climate changes, the ecological niche occupied by *P. fortunei* may face unprecedented challenges and alterations, potentially leading to adjustments in its spatial distribution. Specifically, climate warming may cause shifts in its distribution range, driving it to expand northward or migrate to higher altitudes. For example, Maruthadurai et al. (2024) predicted an increase in the area of suitable habitat for *Neoplocaederus ferrugineus* in the future (2050s and 2070s). Therefore, an in‐depth study of climate change adaptation of *P. fortunei* , which is restricted to Northeast Asia, is of profound significance for predicting its potential invasion risk and developing proactive pest control strategies. Moreover, these findings will provide valuable references for the development of regional pest management strategies in the context of global climate change, helping us to better understand and address the various challenges posed by global environmental change.

### 4.1 Accuracy of the Maximum Entropy Model

The accuracy of SDMs results from the interplay of multiple factors. To construct a high‐precision model, we considered several key aspects, including the quality and quantity of species occurrence data, the selection of environmental variables, and the optimization of software parameters (Wang et al. 2023; Zhang et al. 2024c). In this study, researchers have continuously conducted *P. fortunei* field surveys in multiple provinces and cities in China using handheld GPS since 2013, which significantly enhanced the completeness and reliability of the species' distribution dataset. Compared to existing literature and online public databases, our surveys added 42 new occurrence points, greatly expanding the number of distribution points and revealing a new trend of range extension into central China. These new occurrence records significantly improve the understanding of the species' ecological niche. Additionally, optimizing two key parameters in the MaxEnt model—RM and FCs—improved the model's robustness and predictive capability (Gao et al. 2023). We found that when FC = LQHP and RM = 0.5, delta.AICc = 0, indicating that adjusting these critical parameters significantly improves both the model's predictive performance and its generalizability. The optimized model achieved an average AUC value exceeding 0.96 for predicting *P. fortunei* , demonstrating a high level of accuracy and reliability. The low standard deviation (SD) across multiple replicates indicates model stability and consistency, further enhancing confidence in its predictions. High AUC values are not uncommon in species distribution modeling studies, especially when supported by high‐quality data and rich environmental variables. For example, Liu, Liu, et al. (2024), Liu, Peng, et al. (2024), Liu, Zhao, et al. (2024) use of the MaxEnt model to predict an AUC of 0.980 for *Phenacoccus solenopsis* Tinsley is in agreement with our AUC value, suggesting that MaxEnt has a high predictive accuracy under certain conditions (Liu, Liu, et al. 2024; Liu, Peng, et al. 2024; Liu, Zhao, et al. 2024). Furthermore, our results are consistent with SDM studies of other invasive species. Both *Aleurodicus rugioperculatus* Martin and *Monolepta signata* Olivier exhibit northward expansion under future climate scenarios (Maruthadurai et al. 2023; Liu, Liu, et al. 2024; Liu, Peng, et al. 2024; Liu, Zhao, et al. 2024). These SDM similarities demonstrate the broader applicability of our modeling framework and highlight the critical role of combining high‐quality occurrence data and model optimization in improving the accuracy of invasive species range expansion predictions. Through these comprehensive and in‐depth efforts, we have substantially enhanced the predictive ability of SDMs, allowing for a more precise understanding and prediction of species distribution and ecological responses under diverse climatic conditions.

Although this study effectively predicted potential suitable distribution areas for *P. fortunei* , the uncertainties and limitations of the model results need to be addressed. Firstly, ENMs are extremely dependent on the accuracy and completeness of input data in predicting the potential distribution of species. Any error or bias in the data source will affect the model's identification of the ecological needs of the species, and thus the final prediction results. Secondly, climate models are inherently uncertain, and their projections are limited by factors, such as the setting of future greenhouse gas emission scenarios, differences in model structure, and spatial and temporal resolution. Although these projections can help to reveal macro trends, there may still be deviations from the actual situation at the regional scale. Although anthropogenic data provide the socioeconomic context for analyses, their spatial resolution tends to be coarse and it is difficult to accurately portray disturbance processes at the microlevel, such as urban sprawl, transport, and agricultural activities. The limitations of such data may also affect the assessment of species habitat suitability, especially in areas of rapid urbanization or frequent anthropogenic disturbance. In addition, biological factors such as biological interactions between species, *P. fortunei* 's ability to adapt to environmental changes, and its actual dispersal limits have not been considered in this study, which may affect its true distribution pattern. Future studies could further combine multiple climate models, higher resolution data on human activities, and try to introduce mechanistic models to improve the prediction accuracy. Meanwhile, it is recommended that sensitivity analysis and uncertainty assessment methods be introduced to more systematically analyze the stability and reliability of model results under different assumptions.

### 4.2 Analysis of Environmental Variables

In this study, the main environmental factors affecting *P. fortunei* were analyzed in depth using the Jackknife method (Zhao et al. 2023). The results showed that five climatic variables had key roles in predicting the potential distribution of the species: Bio3 (isothermality), Bio4 (temperature seasonality), Bio11 (mean temperature of coldest quarter), Bio14 (precipitation of driest month), and Bio18 (precipitation of warmest quarter). These factors reflected the changing patterns of temperature and precipitation in the spatial and temporal dimensions, revealing a significant synergistic effect of energy and moisture conditions on the distribution of *P. fortunei* (Santana et al. 2019; Liu, Liu, et al. 2024; Liu, Peng, et al. 2024; Liu, Zhao, et al. 2024). Specifically, higher isothermality (Bio3) usually implies a smaller diurnal temperature difference, which helps to maintain the stability of insect body temperature and favors their metabolic homeostasis and activity continuity. Whereas temperature seasonality (Bio4) is related to the annual temperature difference; too much variation may limit the survival of insects during extreme seasons. Mean temperature of coldest quarter (Bio11) is especially critical for the survival of overwintering adults or larvae and determines the ability of the species to survive in cold environments; lower temperatures may affect its development and reproduction cycle (Yin et al. 2021; Zhang et al. 2023). Meanwhile, precipitation of driest month (Bio14) reflects the water stress level of insects during the dry season, whereas precipitation of warmest quarter (Bio18) influences their water recharge and vegetation growth under high temperature conditions, thus indirectly affecting host availability (Bale 2002). The synergistic effect of these climatic factors constructs the energy and water balance conditions required for the distribution of *P. fortunei* . Furthermore, increasing drought and decreasing precipitation in the context of climate change may reduce the ecological stability of the original habitat, limiting reproductive success and individual survival and encouraging the species to migrate to areas with a relatively mild and humid climate (Wang et al. 2022). In addition, human activities, especially the increase in population density (PD), have also had a significant impact on the distribution of *P. fortunei* . Densely populated areas may provide more vectors and habitat for the species, thus accelerating its spread. It is worth noting that the distribution probability of *P. fortunei* increased with increasing population density (PD), suggesting that human activities may indirectly contribute to its survival and dispersal through a variety of ways. For example, changes in land use (e.g., urban expansion, conversion of agricultural and forested land) may lead to fragmentation of habitat structure, altering regional microclimatic conditions and species migration pathways. At the same time, the development of transport networks may also facilitate passive dispersal (Zhang et al. 2024b). These results emphasize the importance of incorporating human disturbance factors into species distribution projections and risk assessments under future climate scenarios in order to more fully understand potential dispersal mechanisms and ecological adaptation strategies.

### 4.3 Potential Distribution Areas and Displacement

As future climate warming and drying trends intensify, predictions show that, except for the 2070s SSP1‐2.5 scenario, *P. fortunei* 's distribution area is expected to expand to varying degrees in all other scenarios and periods. This phenomenon may reflect *P. fortunei* 's adaptability to temperature and water utilization strategies in response to climate change. For instance, in the context of future global warming and climate drying, *P. fortunei* 's tolerance to higher temperatures allows it to expand its range in warmer environments (Liu, Liu, et al. 2024; Liu, Peng, et al. 2024; Liu, Zhao, et al. 2024). Simultaneously, the species may have evolved efficient water utilization strategies, enabling it to survive and reproduce under drought conditions. Climate change may also offer new ecological niche opportunities for *P. fortunei* , allowing it to occupy previously unsuitable habitats (Ge et al. 2019; Kumar et al. 2022). Additionally, interspecies competition plays a role; under certain future carbon emission scenarios, climate change might reduce the number of competing species, thereby providing *P. fortunei* with a competitive advantage and facilitating its range expansion (Zhang, Dang, et al. 2022; Zhang, Wang, et al. 2022). It is also important to consider how *P. fortunei* might interact with native competitors or predators. These interactions could influence its ability to establish and expand in certain regions, requiring further research to understand the ecological dynamics. However, it is important to note that even though the distribution area may increase in most scenarios, not all regions will offer suitable habitat quality for *P. fortunei* . Some areas may still face habitat degradation or become even less suitable due to climate change (Terblanche et al. 2024). Therefore, the management and control of *P. fortunei* must take into account not only its potential range expansion but also the associated risks to forests and agroecosystems. Moreover, the implementation of pest management strategies requires careful consideration of costs and feasibility, particularly in regions with limited resources for large‐scale control efforts.

By analyzing the potential distribution centers of *P. fortunei* in future climate periods, an interesting and consistent trend is observed: under all carbon emission scenarios, the species shows a tendency to migrate northeastward toward higher latitude regions (Spaak et al. 2021). This indicates that *P. fortunei* possesses unique adaptation strategies to temperature and precipitation pattern changes induced by climate change, seeking more suitable environments at higher latitudes. This migration trend is also closely related to its temperature adaptability, as *P. fortunei* appears to prefer cooler climates, driving it to migrate to higher latitudes, consistent with our analysis of its key environmental factors (Kubelka et al. 2022). Moreover, the impact of climate change on precipitation patterns should not be overlooked. Potential changes in precipitation patterns in future high‐latitude areas may create favorable conditions for the survival and reproduction of the species. Ecological competition and ecological adaptability also play crucial roles in its migration decisions. As climate conditions deteriorate in lower latitude areas, *P. fortunei* may face more intense ecological competition (Mazziotta et al. 2022). Therefore, migrating to higher latitude regions is not only a strategy to avoid competition but also to ensure the survival and reproduction of the population under the pressures of climate change.

### 4.4 Management Implications and Regional Recommendations

Climate change is having a significant impact on the habitat suitability of *P. fortunei* and is expected to drive the expansion of its range, especially in areas of increasing temperatures. As climate change intensifies in the future, *P. fortunei* may expand further beyond its current range to higher latitudes and altitudes, posing greater invasive pressure on agricultural and forestry ecosystems. Therefore, prevention and control strategies for *P. fortunei* need to match the expected trend of climate change, and develop regional, scientifically based prevention and control measures. Priority should be given to strengthening the monitoring and early warning of *P. fortunei* in areas of high suitability, especially in south‐central China, the whole of Japan, and southern South Korea, which may become the key areas for the expansion of *P. fortunei* in the future due to their suitable climatic conditions. For these high‐risk areas, the relevant management authorities should take into account the predictions of climate models and formulate targeted prevention and control strategies, such as increasing the frequency of monitoring, promoting early warning systems, and strengthening public awareness education, in order to detect and respond to the invasion of *P. fortunei* as early as possible. In addition, in areas where climate change is more significant, measures such as ecological restoration and protective forest construction are recommended to reduce the habitat space for *P. fortunei* . For example, in areas with low forest cover, consideration should be given to enhancing the resistance of ecosystems to invasion through afforestation and restoration of degraded ecosystems, so as to reduce the expansion of suitable habitat for *P. fortunei* at source. At the same time, the spread of the pest can be reduced and the ecosystem protected through the implementation of integrated pest management strategies that combine biological, chemical, and cultural controls. These regional management strategies can not only effectively control the expansion of *P. fortunei* , but also provide lessons and references for pest control in other regions in the context of global climate change, ensuring sustainable development of agriculture and forestry.

## 5 Conclusions

Through long‐term field surveys and extensive literature review, this study systematically collected the latest distribution data on *P. fortunei* . By integrating bioclimatic and anthropogenic factors and utilizing an optimized MaxEnt niche model, we conducted an in‐depth and comprehensive assessment of the potential distribution areas and dynamic changes of *P. fortunei* in Northeast Asia. The results indicate that temperature (Bio3, Bio4, and Bio9), precipitation (Bio14 and Bio18), and human activities (PD) together influence the potential distribution of *P. fortunei* . Under the current climate conditions, *P. fortunei* is primarily concentrated in China, North Korea, South Korea, Japan, Vietnam, Bhutan, India, and Nepal. Model predictions suggest that under future climate scenarios, the potential habitat of *P. fortunei* is expected to expand to central and western China, Japan's Hokkaido Island, Russia's Sakhalin Island, Vietnam, Myanmar, India, Nepal, and Bangladesh. Furthermore, the potential distribution center of *P. fortunei* is projected to shift toward higher latitude regions under future climate conditions. This spatial migration may facilitate the species' spread and enhance its ability to adapt to new climatic environments. In summary, this study provides robust scientific support for developing management strategies for *P. fortunei* as a pest in the region, helping us more effectively address the challenges posed by global climate change. This study aims to inform effective pest management strategies that help reduce the risk of biological invasions while preserving ecosystem integrity and function.

## Author Contributions

**Ping Wang:** conceptualization (equal), data curation (equal), funding acquisition (equal), investigation (equal), methodology (equal), validation (equal), visualization (equal), writing – original draft (equal), writing – review and editing (equal). **Liang Zhang:** conceptualization (equal), formal analysis (equal), methodology (equal), software (equal), validation (equal), visualization (equal), writing – original draft (equal), writing – review and editing (equal). **Jie Li:** data curation (equal), investigation (equal), validation (equal). **Chaokun Yang:** formal analysis (equal). **Guanglin Xie:** investigation (equal), supervision (equal). **Wenkai Wang:** funding acquisition (equal), supervision (equal), writing – review and editing (equal).

## Conflicts of Interest

The authors declare no conflicts of interest.

## Supporting information


**Figure S1.**Correlation among the 22 bioclimatic variables.
**Figure S2.** Omission rates for different cumulative thresholds in the MaxEnt model.
**Table S1.** Correlation analysis and screening of 22 climate variables.

## Data Availability

All the required data are uploaded as Supporting Information.
