# Supplementary material for: Using Ecological Modeling to Study the Response of Distribution Dynamics of Paraglenea fortunei (Coleoptera: Cerambycidae) to Human Activities and Climate Change to in Northeast Asia
Source: Ecol Evol. 2025 Jul 8;15(7):e71782. doi: 10.1002/ece3.71782 (PMC12237826; doi:10.1002/ece3.71782)
Supplement: Supplementary file 1 — Figure S1.Correlation among the 22 bioclimatic variables. Figure S2. Omission rates for different cumulative thresholds in the MaxEnt model. Table S1. Correlation analysis and screening of 22 climate variables. [file ECE3-15-e71782-s001.docx]

**Using ecological modelling to study the response of human activities and climate change to the distribution dynamics of *Paraglenea fortunei* (Coleoptera: Cerambycidae) in Northeast Asia**

**Supplementary data:**


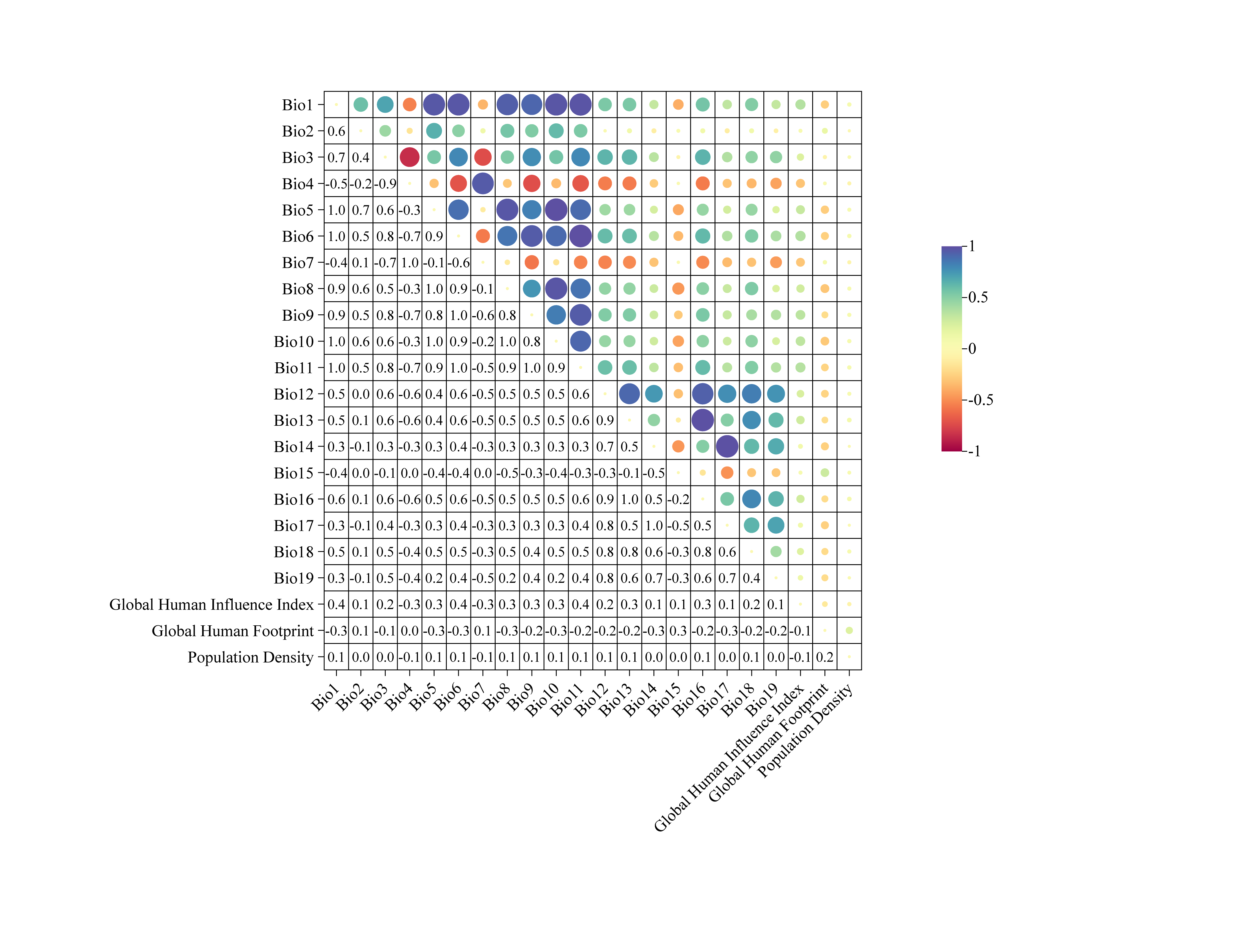


Fig. S1. Correlation among the 22 bioclimatic variables.


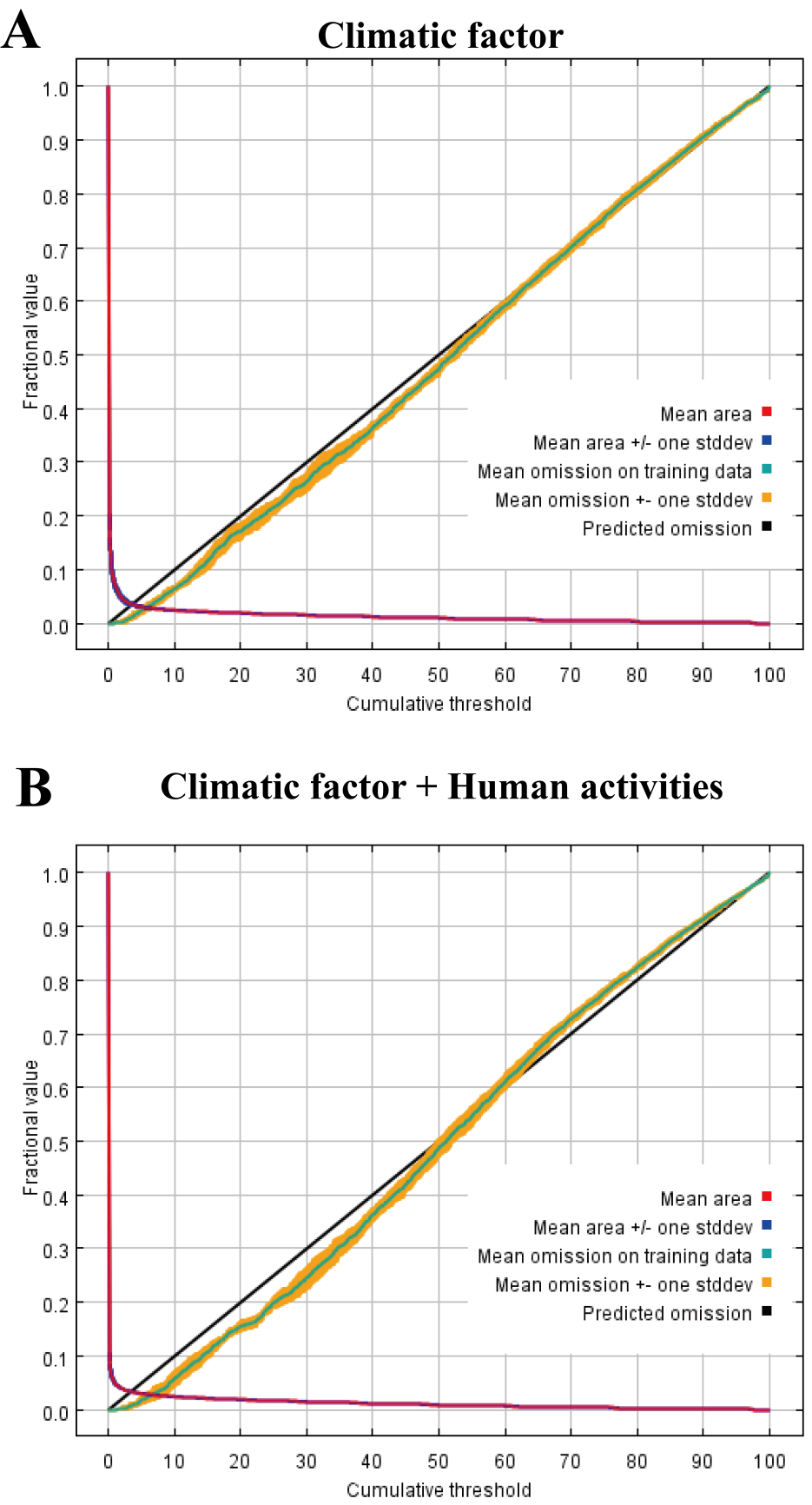


Fig. S2. Omission rates for different cumulative thresholds in the MaxEnt model. Note: (A) Climate factor; (B) Climate factor + human activities.

Table S1. Correlation analysis and screening of 22 climate variables.

| Abbreviation | Environmental variables | Operation (\|*r*\| > 0.9) |
| --- | --- | --- |
| Bio1 | Annual mean temperature (◦C) | Eliminate |
| Bio2 | Mean diurnal range (◦C) | Retain |
| Bio3 | Isothermality | Retain |
| Bio4 | Temperature seasonality | Retain |
| Bio5 | Maximum temp of warmest month (◦C) | Eliminate |
| Bio6 | Minimum temp of coldest month (◦C) | Eliminate |
| Bio7 | Temperature annual range (◦C) | Eliminate |
| Bio8 | Mean temp of wettest quarter (◦C) | Eliminate |
| Bio9 | Mean temp of driest quarter (◦C) | Eliminate |
| Bio10 | Mean temp of warmest quarter (◦C) | Retain |
| Bio11 | Mean temp of coldest quarter (◦C) | Retain |
| Bio12 | Annual precipitation (mm) | Eliminate |
| Bio13 | Precipitation of wettest month (mm) | Eliminate |
| Bio14 | Precipitation of driest month (mm) | Retain |
| Bio15 | Precipitation seasonality (mm) | Retain |
| Bio16 | Precipitation of wettest quarter (mm) | Eliminate |
| Bio17 | Precipitation of driest quarter (mm) | Eliminate |
| Bio18 | Precipitation of warmest quarter (mm) | Retain |
| Bio19 | Precipitation of coldest quarter (mm) | Eliminate |
| GHII | Global human influence index | Retain |
| GHF | Global human footprint | Retain |
| PD | Population density | Retain |
